# Higher Relative Abundance of Glomeromycota in Topsoil Under *Caragana korshinskii* Sand-Fixation Patterns in the Mu Us Sandy Land

**DOI:** 10.3390/plants15213338

**Published:** 2026-10-28

**Authors:** Wangsuo Liu, Kaiyang Qiu, Xiaoli Zhang, Yanming Zhang, Junfeng Wang, Xinning Han, Wenfei Dong

**Affiliations:** 1 School of Ecological Environment and Chemical Engineering, Hetao College, Bayannur 015000, China; 15809607727@163.com (X.Z.); zym18gz@163.com (Y.Z.); wangjunfengww@126.com (J.W.); 2 Urat Desert-Grassland Observation and Research Station, Inner Mongolia Autonomous Region, Bayannur 015000, China; 3 School of Forestry and Grassland Science, Ningxia University, Yinchuan 750021, China; 4 Ningxia Research Center of Engineering and Technology for Prataculture and Animal Husbandry, Yinchuan 750021, China; 5 Breeding Base for State Key Laboratory of Land Degradation and Ecological Restoration of Northwest China, Yinchuan 750021, China; 6 College of Ecology and Environment, Ningxia University, Yinchuan 750021, China; 7 College of Resources Environment and Life Sciences, Ningxia Normal University, Guyuan 756000, China; hanxinning@163.com; 8 College of Chemistry and Chemical Engineering, Ningxia Normal University, Guyuan 756000, China; dwf19995362248@163.com

**Keywords:** *Caragana korshinskii*, sand fixation patterns, Glomeromycota, arbuscular mycorrhizal fungi, desertification reversal

## Abstract

Revegetation with native shrubs is central to combating desertification, yet whether and how plants reshape soil microbial communities in ways that may contribute to sand fixation remains poorly understood. Here, we compared three sand-fixation patterns, pure *Caragana korshinskii* (Cak), pure *Corethrodendron fruticosum* var. *mongolicum* (Cof), and mixed *C. korshinskii* with *C. scoparium* (CakCos), in the Mu Us Sandy Land using ITS high-throughput sequencing. We focused on the relative sequence abundance of Glomeromycota, a phylum consisting almost exclusively of arbuscular mycorrhizal fungi (AMF), as an indicator of potential AMF-related taxa. The relative abundance of Glomeromycota was 2.3–3.1 times higher in Cak and CakCos than in Cof, with *Glomus* and *Septoglomus* as the core enriched genera. Correlation and redundancy analyses identified soil moisture, total carbon, electrical conductivity, and carbon-to-nitrogen (C:N) ratio as key environmental factors associated with this pattern. FUNGuild-based bioinformatic predictions further suggested that *C. korshinskii* was associated with a shift in the fungal functional balance toward the AM guild while relative to saprotrophic and potentially pathogenic guilds. These findings are correlative and do not establish causation, but they suggest that *C. korshinskii* may act as an environmental filter that creates nutrient-rich “resource islands” and may be associated with the enrichment of Glomeromycota. Our findings contribute to the development of dryland restoration theory and provide a valuable microbial perspective for optimizing shrub selection in desertification control.

## 1. Introduction

Desertification affects over 40% of the global land area and threatens ecosystem services and human livelihoods in arid and semi-arid regions [1]. In Northern China, the Mu Us Sandy Land exemplifies severe wind erosion and dune mobility, where reversing degradation requires not only physical stabilization but also the recovery of functional plant–soil systems [2]. Among revegetation approaches, planting native leguminous shrubs has emerged as a sustainable strategy, as these species can simultaneously stabilize sand, enrich soil nitrogen, and improve microclimatic conditions [3]. While engineering structures like straw checkerboards provide initial surface stabilization, long-term restoration relies on establishing resilient vegetation that can initiate soil recovery.

*Caragana korshinskii* Kom., a perennial leguminous shrub endemic to Northern China, has been widely deployed in sand-fixation projects due to its drought tolerance, deep root system, and ability to form symbiotic associations with nitrogen-fixing rhizobia [4,5]. Previous studies have documented its “nurse plant” effects, including reduced soil evaporation, increased litter input, and enhanced nutrient accumulation under its canopy. This phenomenon is known as “resource island” formation [6,7]. However, most research has focused on aboveground benefits or bulk soil chemistry, while the belowground microbial mechanisms that may underpin its restoration success remain underexplored.

Soil fungi, particularly arbuscular mycorrhizal fungi (AMF) within the phylum Glomeromycota, play critical roles in ecosystem functioning. Glomeromycota consist almost exclusively of AMF, which form mutualistic symbioses with the majority of terrestrial plants [8]. AMF enhance plant nutrient (especially P and N) and water uptake, produce glomalin to bind soil particles into stable aggregates, and can suppress soil-borne pathogens [9,10,11]. In desert ecosystems, AMF are recognized as key facilitators of plant establishment and soil stabilization, yet their response to different shrub species and their functional implications for sand fixation are rarely investigated at the community level in bulk topsoil. Because of this direct link to aggregate formation and sand stabilization, we focused on the fungal compartment, particularly Glomeromycota. Bacteria and archaea are also important in nutrient cycling, but their responses to these same sand-fixation patterns have been addressed in our previous work [12]. The present study therefore provides a complementary fungal perspective. Most previous field studies targeting AMF under *C. korshinskii* focused on rhizosphere compartments; by contrast, we focused on non-rhizosphere bulk topsoil to characterize landscape-level fungal signals across sand-fixation shrub configurations, including pure and mixed shrub stands.

In this study, we conducted an observational comparison among three representative sand-fixation patterns in the Baijitan National Nature Reserve (Mu Us Sandy Land): pure *C. korshinskii* (Cak), pure *Corethrodendron fruticosum* var. *mongolicum* (Cof), and mixed *C. korshinskii* with *C. scoparium* (CakCos). Our aim was to elucidate whether and how *C. korshinskii* influences topsoil fungal communities, with a particular focus on Glomeromycota. We tested three hypotheses: (H1) sand-fixation patterns involving *C. korshinskii* would be associated with improved topsoil physicochemical properties compared with the non-*C. korshinskii* pattern (Cof); (H2) stands containing *C. korshinskii* would be associated with higher relative sequence abundance of Glomeromycota, but not with overall fungal α-diversity; and (H3) the relative abundance of Glomeromycota would be correlated with soil factors associated with *C. korshinskii*, and predicted functional guilds would differ among treatments. We emphasize that this study is observational and cannot establish causation, true microbial recruitment, or functional symbiotic activity. By integrating soil physicochemical analyses, high-throughput ITS sequencing, and functional guild predictions, this research seeks to generate hypotheses about the microbial patterns underlying *C. korshinskii*’s success in sand-fixation ecosystems and to provide a scientific basis for optimizing desertification control strategies.

## 2. Results

### 2.1. Changes in Physicochemical Properties in Topsoil Under Sand-Fixation Patterns Involving C. korshinskii

**Figure 1:**
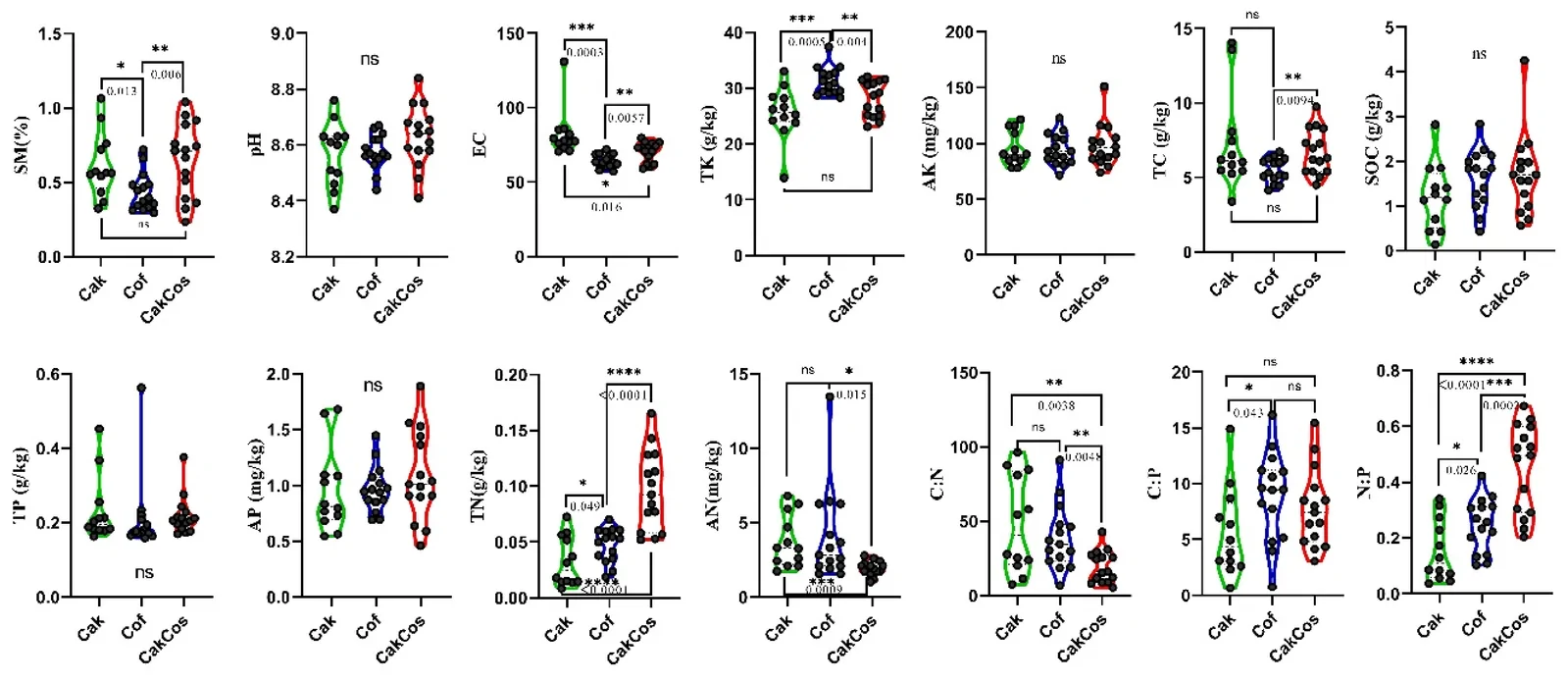
Changes in physicochemical properties of topsoil under sand-fixation patterns involving *Caragana korshinskii*. SM: soil moisture; EC: electrical conductivity; TN: total nitrogen; TC: total carbon; SOC: soil organic carbon; TK: total potassium; AK: available potassium; TP: total phosphorus; AP: available phosphorus; C:N, C:P, N:P represent SOC:TN, SOC:TP, TN:TP stoichiometric ratios, respectively. Statistical analyses use Kruskal–Wallis test followed by Dunn’s post hoc test for multiple comparisons and Mann–Whitney U-tests for pairwise contrasts. ns: non-significant; * *p* < 0.05, ** *p* < 0.01, *** *p* < 0.001, **** *p* < 0.0001.

The soil physical and chemical properties at a depth of 0–10 cm significantly differed among the three sand-fixing patterns (Figure 1). Both Cak and CakCos are sand-fixation patterns involving *C. korshinskii*. The content of soil moisture (SM), electrical conductivity (EC), and total nitrogen (TN) was significantly higher in Cak and CakCos than in Cof, while total potassium (TK) and available nitrogen (AN) showed the opposite trend. Total carbon (TC) was significantly higher in CakCos, indicating that it had an accumulation advantage in the mixed forest sand-fixation pattern. The trends observed in the C:N ratio (SOC/TN) and N:P ratio (TN/TP) were opposite, whereas the C:P ratio (SOC/TP) was relatively higher in Cof. Particularly, N:P ratio and TN content were significantly elevated in CakCos, consistent with a contribution of leguminous shrub mixed sand-fixation forest to nitrogen accumulation in the topsoil.

### 2.2. Changes in Fungal Communities in Topsoil Under Sand-Fixation Patterns Involving Caragana korshinskii

**Figure 2:**
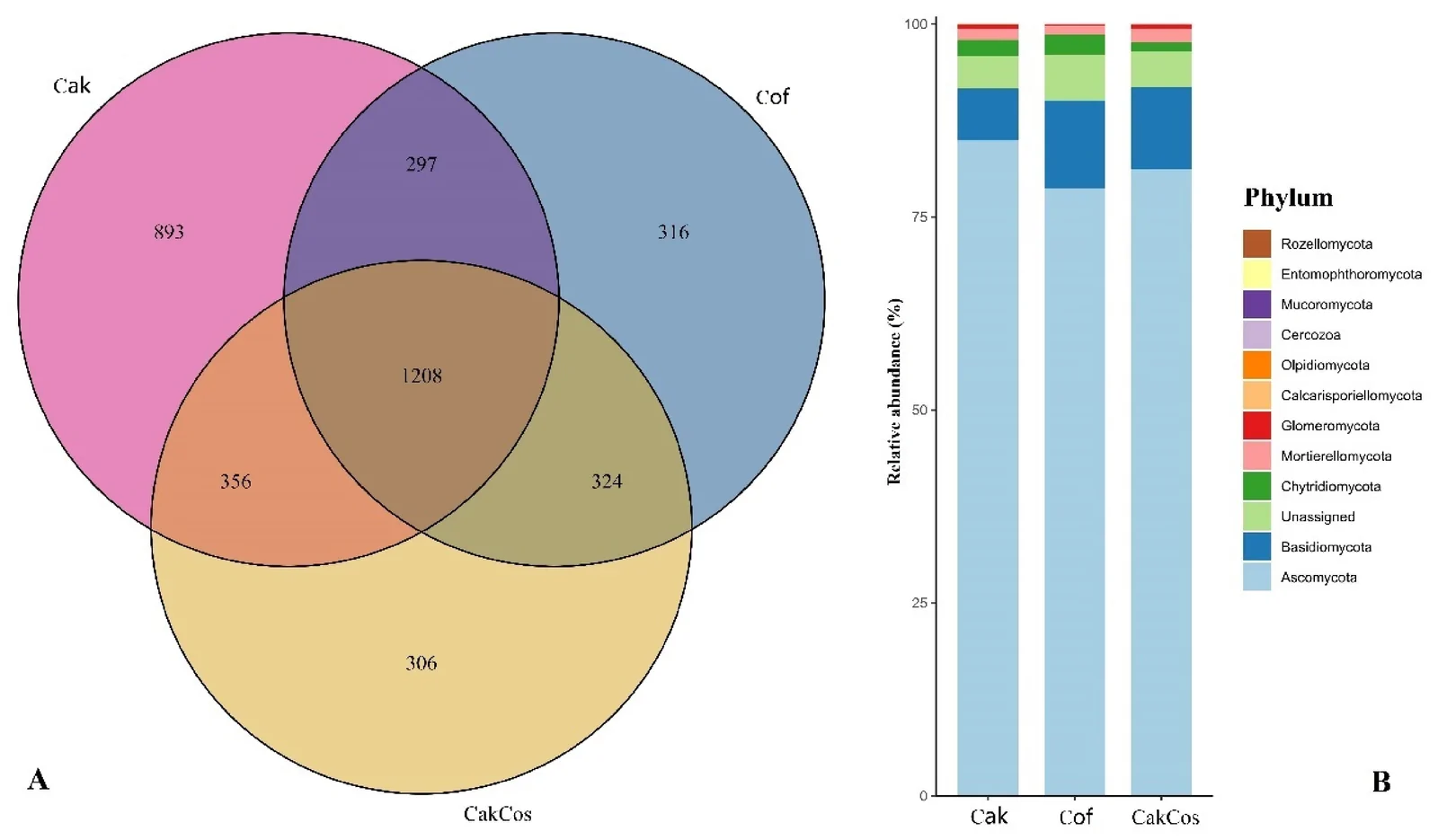
Characteristics of fungal community structure in topsoil under sand-fixation patterns involving *C. korshinskii*. (**A**) Venn diagram showing shared and unique operational taxonomic unit (OTU) counts across three sand-fixation patterns. (**B**) Fungal community composition at the phylum level, expressed as relative sequence abundance.

After quality control, filtration and splicing, a total of 4,828,451 clean reads were obtained from 43 topsoil samples of the three sand-fixing patterns by ITS gene sequencing. The number of clean reads ranged from 43,244 to 190,263, with an average of 112,290 (Supplementary Table S1). The Venn diagram illustrates a total of 1208 operational taxonomic units (OTUs) shared among the three sand-fixing patterns, while significant variations are observed in the unique OTUs. Specifically, Cak exhibits the highest number of unique OTUs with 893, whereas Cof and CakCos have 316 and 306 unique OTUs respectively (Figure 2A). The dominant fungal phyla in each pattern were Ascomycota, Basidiomycota, Unassigned, Chytridiomycota, Mortierellomyctoa, and Glomeromycota, in which Ascomycota and Basidiomycota accounted for 81.6% and 9.6% of the phylum, respectively (Figure 2B). The dominant fungal genera at the genus level included Unassigned, *Alternaria*, *knufia*, *Chaetosphaeronema*, *Preussia*, *unclassified_Pleosporales*, *Tulostoma*, and *Paraphoma*, etc., with the Unassigned groups comprising the majority proportion (Supplementary Figure S1A). There was no significant change in alpha diversity (Shannon, Simpson, Chao1, and ACE indices) of the fungal community among the three sand-fixation patterns (Supplementary Figure S1B), indicating that *C. korshinskii* did not alter overall fungal species richness or evenness. This pattern of stable overall α-diversity alongside a shift in a specific taxonomic group (Glomeromycota) is consistent with a selective rather than whole-community response.

**Figure 3:**
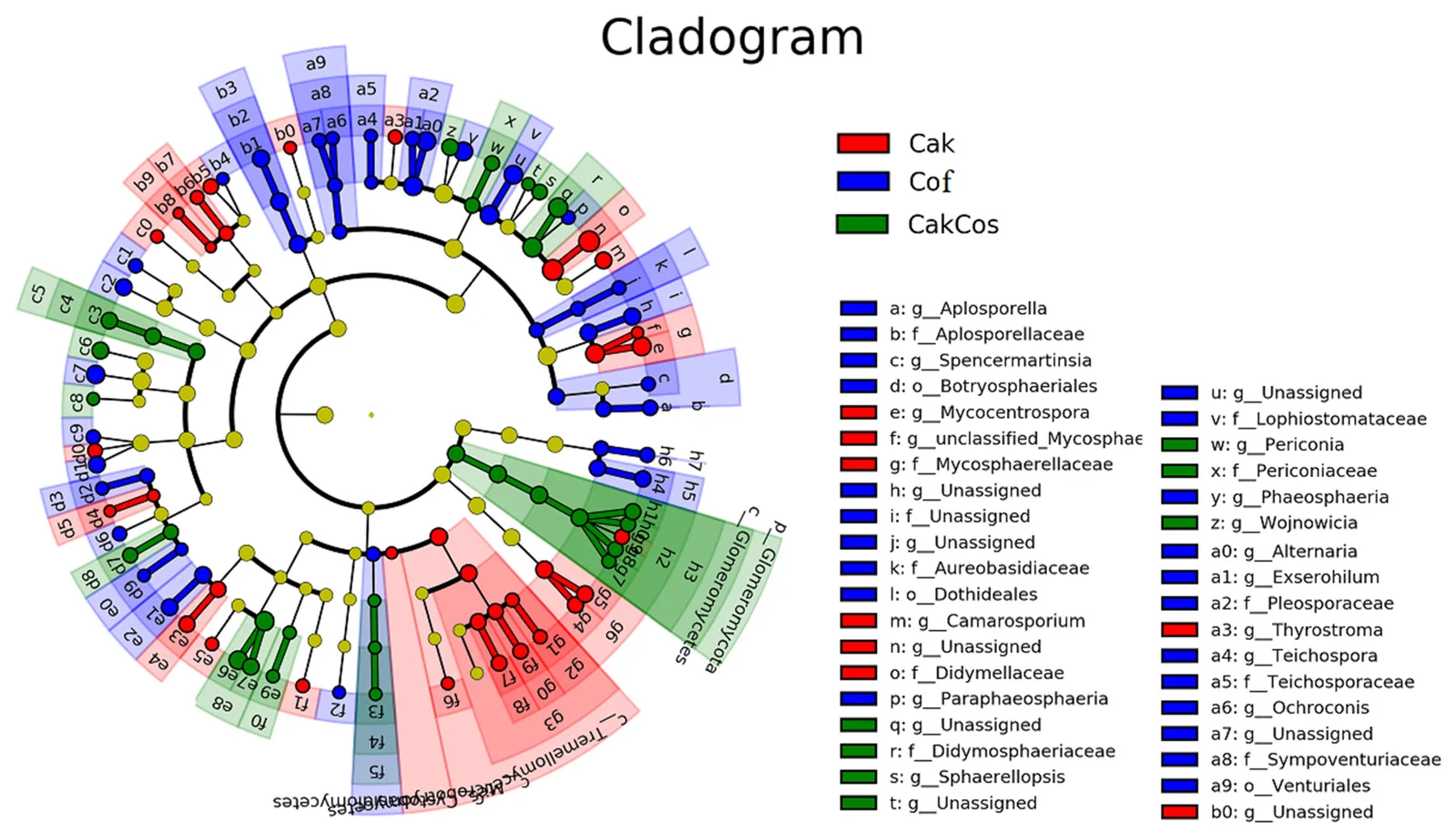
Cladogram based on LEfSe analysis showing differentially enriched fungal taxa under sand-fixation patterns involving *C. korshinskii* (|LDA| > 2 and *p* < 0.05). Only nodes related to Glomeromycota are labeled; the exploratory cladogram without *p*-value filtering and the complete labeling are provided in Supplementary Figure S3. Node colors correspond to treatment groups; colored branches denote taxa with significantly higher relative-sequence abundance in the corresponding treatment.

In order to investigate the difference taxon of topsoil fungi between the sand-fixation patterns involving *C. korshinskii* and the without *C. korshinskii* treatment, the LDA score (LDA > 2 and *p* < 0.05) of the screening biomarkers was illustrated by the histograms; the length of each bar means the degree of differences (Supplementary Figure S2). The taxonomic and phylogenetic hierarchy was described by the statistically filtered cladogram (Figure 3; |LDA| > 2 and *p* < 0.05). The exploratory cladogram without *p*-value filtering is provided in Supplementary Figure S3. Most of the fungal OTUs belonged to Ascomycota, Basidiomycota, and Chytridiomycota. However, in sand-fixation patterns involving *C. korshinskii* (Cak and CakCos), Glomeromycota showed a significantly higher relative abundance in the topsoil.

**Figure 4:**
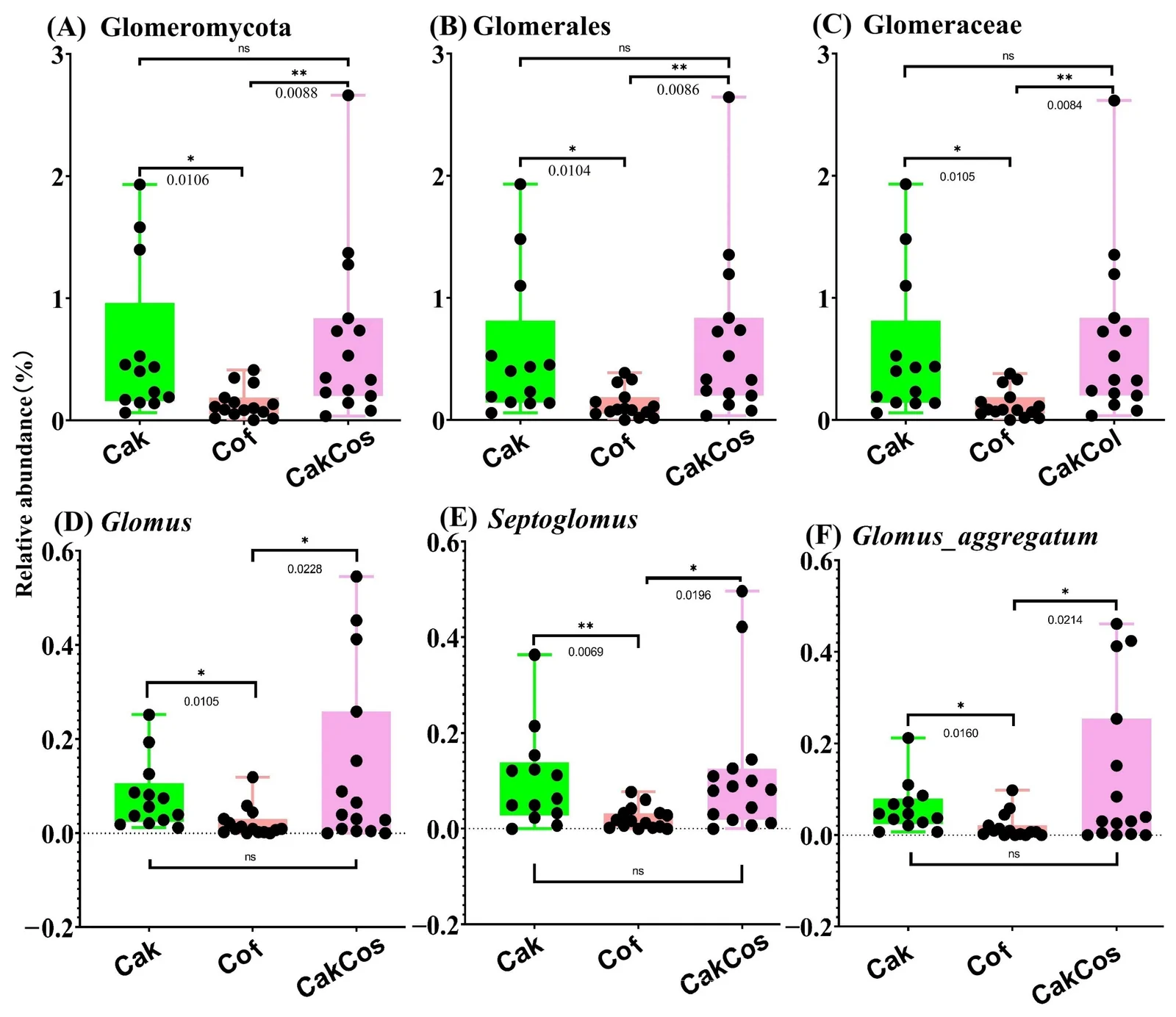
Differences in fungal relative-sequence abundance across hierarchical taxonomic levels in topsoil under sand-fixation patterns involving *C. korshinskii*. Only subpanels directly related to Glomeromycota are shown: phylum (**A**), order (**B**), family (**C**), genus (**D**,**E**), and species (**F**). The complete set of comparisons across all taxonomic levels is provided in Supplementary Figure S4. Statistical significance markers: ns: non-significant; * *p* < 0.05, ** *p* < 0.01.

By comparing the differences at the phylum, order, family, genus, and species levels, Glomeromycota consistently showed higher relative abundance in Cak and CakCos than in Cof (Figure 4A–F). Other fungal groups also differed among treatments, including Chytridiomycota, Botryosphaeriales, Pleosporaceae, *Fusarium*, and *Thyrostroma*; the complete set of comparisons across all taxonomic levels is provided in Supplementary Figure S4. In general, we observed a particularly intriguing phenomenon in the sand-fixation patterns involving *C. korshinskii*: although the abundance of Glomeromycota was lower than that of Ascomycota and other fungal groups, Glomeromycota still tended to show higher relative abundance in sandy land. These results indicate that *Caragana* presence is associated with higher relative abundance of Glomeromycota in the topsoil, but they do not demonstrate colonization or selective recruitment.

**Figure 5:**
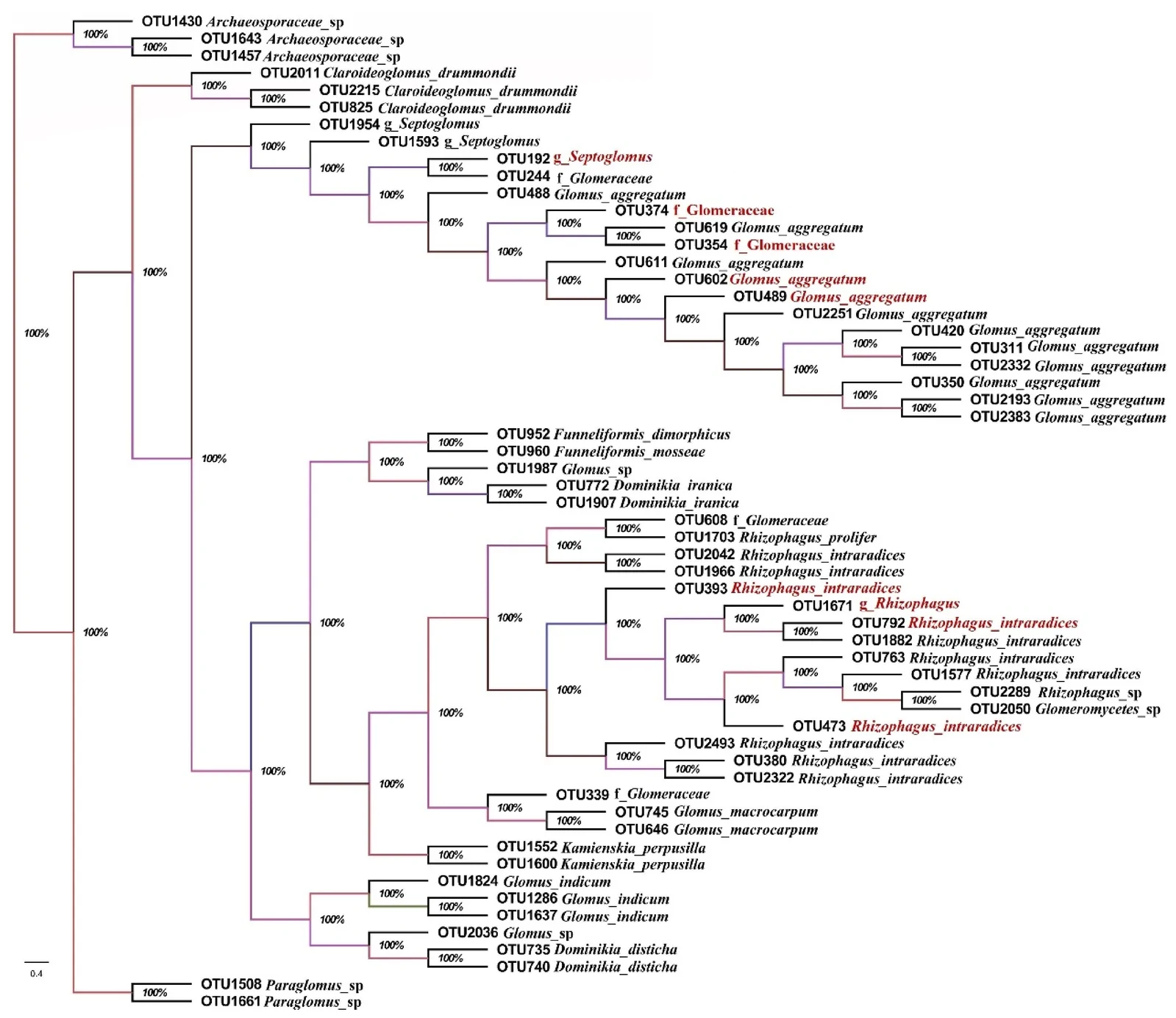
Maximum-likelihood phylogenetic tree of Glomeromycota operational taxonomic units (OTUs) retrieved from topsoil under three shrub sand-fixation patterns. Nearly all bootstrap support values equal 100%, caused by high similarity among these AM fungal amplicon sequences. The tree is constructed in MEGA X with 1000 bootstrap replicates following MAFFT multiple sequence alignment. The red-colored species represent species with extremely significant differences (*p* < 0.001).

To further describe the diversity of Glomeromycota in the sand-fixation pattern involving *C. korshinskii*, we constructed a species-level phylogenetic tree by comparing DNA sequences of OTUs from Glomeromycota members (Figure 5). The results revealed the presence of 58 OTUs representing 16 species, which exhibited two major branches on the evolutionary tree, one identified as *Archaeosporaceae* sp., and the others were grouped together in another cluster. Among them, *Glomus* was found to be the most abundant species, followed by *Rhizophagus*, and these two species showed a close relationship with each other. This indicates that *Glomus* and *Rhizophagus* are the predominant Glomeromycota taxa.

### 2.3. Relationship Between Topsoil Fungal Communities and Soil Physicochemical Properties

**Figure 6:**
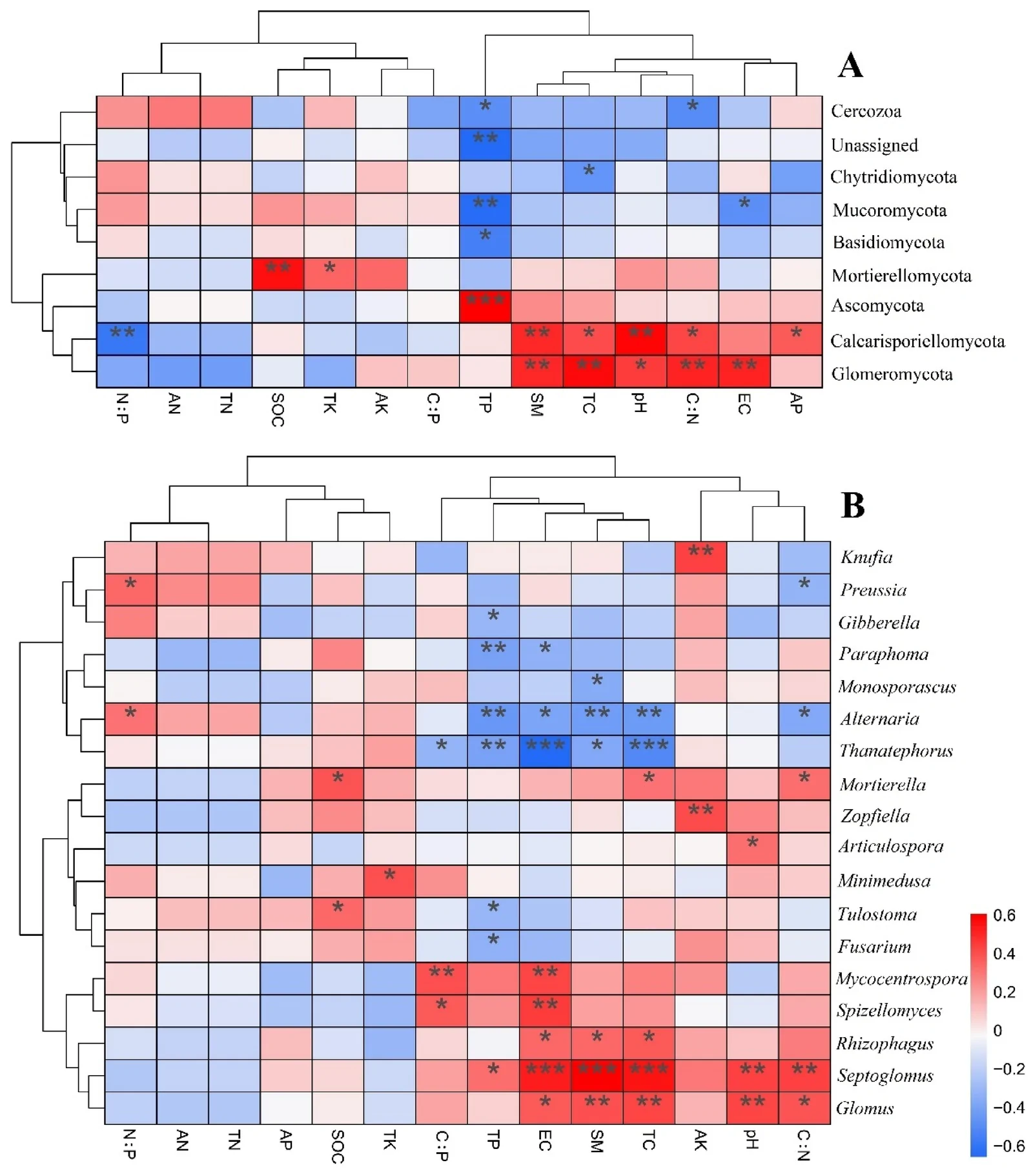
Spearman rank-order correlation heatmaps between fungal community relative-sequence abundance and soil environmental factors in topsoil. (**A**) Phylum-level fungal taxa. (**B**) Genus-level fungal taxa. * *p* < 0.05, ** *p* < 0.01, *** *p* < 0.001.

The correlation heatmap was generated to elucidate the relationship between soil surface fungal communities (phylum-level and genus-level) and soil environmental factors under the sand-fixation pattern. The results revealed significant positive correlations between Glomeromycota and soil environmental factors including SM, TC, EC, C:N ratio, and pH. Similarly, Calcarisporiellomycota also exhibited significant positive correlations with these soil factors, while Mortierellomycota demonstrated a significant positive correlation with SOC and TK. However, the dominant Ascomycota showed only a positive correlation with TP (Figure 6A). It was found that soil environmental factors were closely associated with specific genera from Glomeromycota, such as *Septoglomus* displayed significant positive correlations with EC, SM, TC, pH, C:N ratio, and TP, while *Glomus* also performed significant positive correlations with SM, TC, pH, C:N ratio, and EC (Figure 6B). These findings reveal that the Glomeromycota groups of fungi in sand-fixing topsoils are strongly influenced by crucial soil environmental factors such as SM, EC, TC, C:N ratio, and pH, which play an important role in shaping these fungal communities.

### 2.4. Comparison of Functional Prediction of Fungal Communities in Topsoil

**Figure 7:**
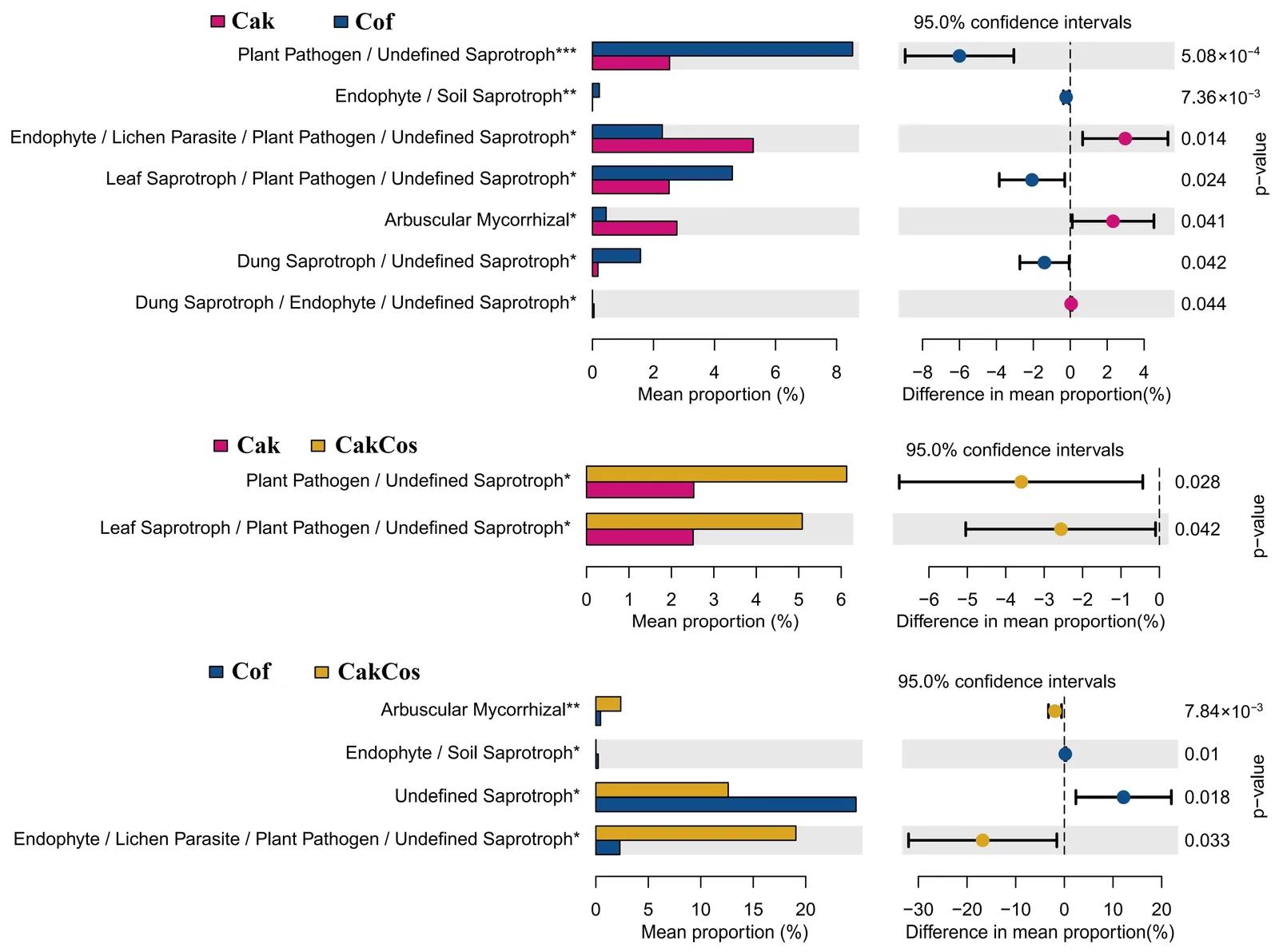
Comparisons of FUNGuild-based bioinformatic predictions of fungal functional-group proportions among sand-fixation patterns. All functional outputs are bioinformatic predictions and not direct measurements of in situ fungal function. Statistical significance markers: ns: non-significant; * *p* < 0.05, ** *p* < 0.01, *** *p* < 0.001.

Based on the FUNGuild database, we performed bioinformatic predictions of fungal functional guilds among the sand-fixation patterns. These predictions are not direct measurements of in situ fungal function. The predicted guild abundances indicated that soil fungi primarily belonged to saprophytic functional groups (Figure 7). Among the comparison between Cak and Cof, the predicted abundances of Plant Pathogen/Undefined Saprotroph (PUS), Endophyte/Soil Saprotroph (ESS), Leaf Saprotroph/Plant Pathogen/Undefined Saprotroph (LPUS), Dung Saprotroph/Undefined Saprotroph (DUS) were significantly higher in Cof, whereas the predicted abundances of Endophyte/Lichen Parasite/Plant Pathogen/Undefined Saprotroph (ELPUS), Arbuscular Mycorrhizal (AM), and Dung Saprotroph/Endophyte/Undefined Saprotroph (DEUS) were significantly higher in Cak. Among the comparison between Cak and CakCos patterns, the predicted abundances of PUS and LPUS were significantly higher in CakCos than in Cak. Furthermore, in the comparison between Cof and CakCos patterns, the predicted abundances of AM and ELPUS were notably higher in CakCos than in Cof; however, the predicted abundances of EES and Undefined Saprotroph (US) were significantly higher in Cof (Figure 7). In particular, the predicted abundance of AM was directly associated with Glomeromycota, suggesting that the sand-fixation patterns involving *C. korshinskii* were associated with a higher predicted abundance of the AM guild in the surface sandy land. However, these FUNGuild outputs are bioinformatic predictions and do not constitute direct measurements of mutualism, pathogen suppression, or ecosystem function.

**Figure 8:**
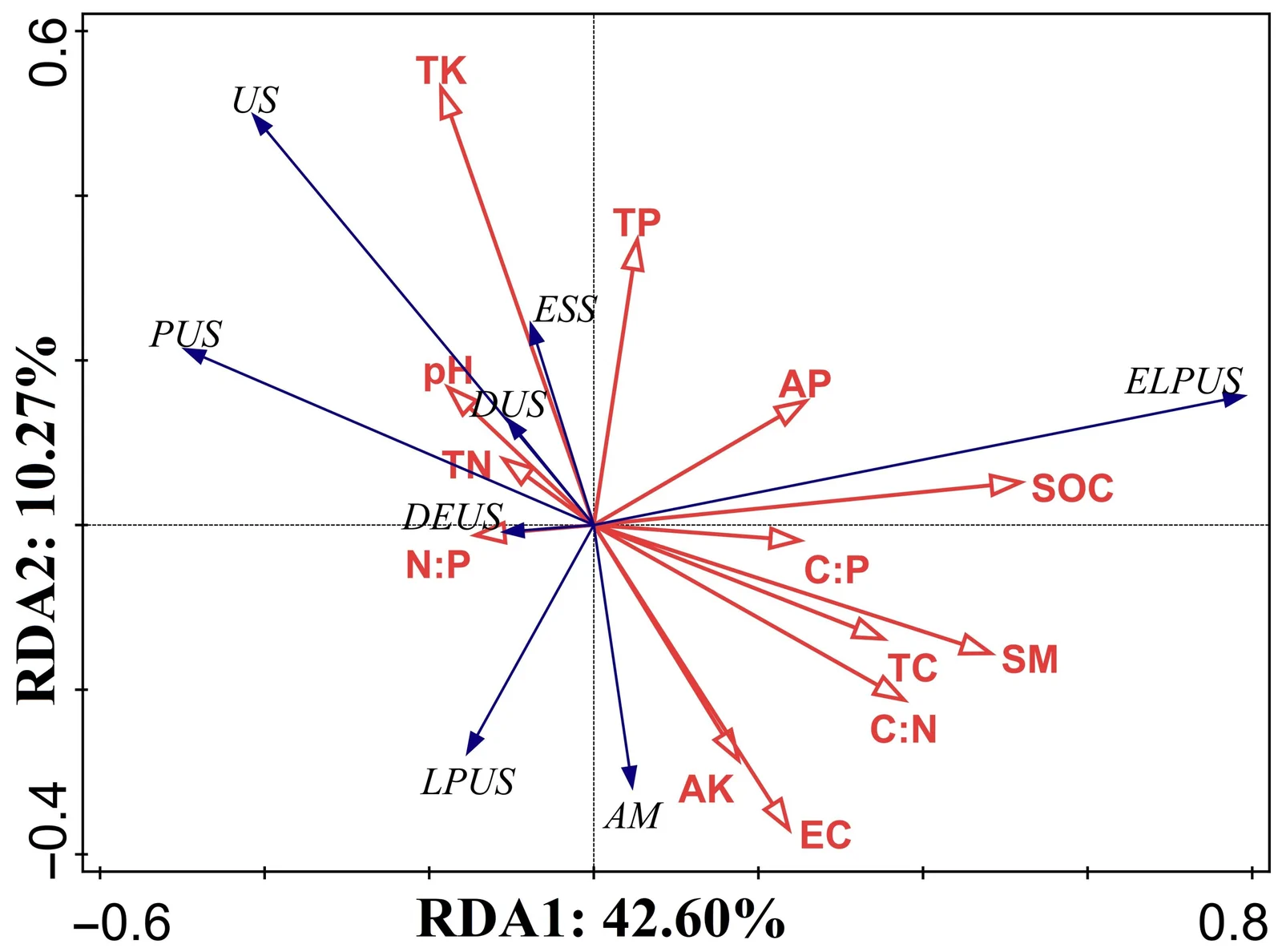
Redundancy analysis (RDA) showing relationships between soil physicochemical properties (red arrows) and FUNGuild-predicted fungal functional groups (blue arrows). Functional-group data were Hellinger-transformed before analysis. RDA1 and RDA2 explain 42.60% and 10.27% of total variation, respectively. Guild abbreviations: US, Undefined Saprotroph; PUS, Plant Pathogen/Undefined Saprotroph; LPUS, Leaf Saprotroph/Plant Pathogen/Undefined Saprotroph; ESS, Endophyte/Soil Saprotroph; ELPUS, Endophyte/Lichen Parasite/Plant Pathogen/Undefined Saprotroph; DUS, Dung Saprotroph/Undefined Saprotroph; DEUS, Dung Saprotroph/Endophyte/Undefined Saprotroph; and AM, Arbuscular Mycorrhizal.

The redundancy analysis revealed a significant correlation between the functional groups of topsoil fungi and soil environmental factors. RDA1 and 2 explained 42.60% and 10.27%, respectively, and accounted for 52.87% of the variation in total; the soil factors such as SOC, C:P, TK, and SM had significant effects. Combined with the study results in Figure 8, it can be found that AK, EC, C:N ratio, TC, and SM exert significant influences on Glomeromycota associated with AM, with the only difference being that AM is affected by AK, not pH value. In a comprehensive analysis, we found that the soil EC, C:N ratio, TC, and SM are crucial soil environmental factors influencing the abundance of both Glomeromycota as well as its functional taxa (AM).

## 3. Discussion

### 3.1. C. korshinskii as a Keystone Species in Shaping Soil Properties

The restoration of degraded ecosystems, particularly in arid and semi-arid regions threatened by desertification, is a global environmental challenge. The first and most fundamental observation of our study is the significant alteration of topsoil physicochemical properties by *C. korshinskii*. Compared to the control shrubland without *C. korshinskii* (Cof), both the pure (Cak) and mixed (CakCos) stands involving *C. korshinskii* led to marked increases in soil moisture (SM), electrical conductivity (EC), total nitrogen (TN), and total carbon (TC) (Figure 1). These changes are not random but form a coherent picture of improved soil health and fertility. The increase in SM can be attributed to the well-documented “nurse plant” effect of desert shrubs [6,7]. The canopy of *C. korshinskii* reduces direct solar radiation and wind speed at the soil surface, thereby lowering evaporation rates. Simultaneously, its complex root system creates macropores that enhance water infiltration during rare precipitation events. Furthermore, research has revealed that *C. korshinskii* possesses specialized leaf trichomes capable of absorbing dew, a potential additional water source that indirectly benefits the rhizosphere soil moisture [5].

The elevated levels of TN and TC are directly linked to the nitrogen-fixing capability of *C. korshinskii* as a leguminous shrub. Through symbiotic relationships with rhizobia in root nodules, *C. korshinskii* can convert atmospheric N_2_ into biologically available ammonia, enriching the soil nitrogen pool [4]. This input of “new” nitrogen is a critical ecosystem service in N-limited desert environments. The increased TN, in turn, drives the observed changes in ecological stoichiometry, particularly the significantly higher N:P ratio in the mixed stand CakCos. This suggests that *C. korshinskii*, especially when paired with another species, can alleviate nitrogen constraints more effectively than other shrubs. The accumulation of TC and SOC is a consequence of multiple processes: root exudates, root turnover and decomposition, and the accumulation of organic matter derived from leaf litter [13,14,15]. The “resource island” effect, where nutrients concentrate under shrub canopies, is a well-known phenomenon in arid lands [16,17,18], and our results strongly confirm that *C. korshinskii* is a highly effective creator of such islands. The increase in EC, often associated with higher solute concentration, likely reflects the accumulation of ions from decomposed organic matter and root exudates, further signifying enhanced biological activity and nutrient cycling in the rhizosphere of *C. korshinskii*. In summary, the establishment of *C. korshinskii* fundamentally reshapes the soil environment, creating a more mesic and nutrient-rich niche that is conducive to supporting a more diverse and functionally distinct microbial community [4].

### 3.2. Glomeromycota Enrichment as a Key Microbial Driver in Caragana korshinskii-Mediated Restoration of Sandy Ecosystems

Long-term revegetation of sandy land has been recognized as essential for shaping diverse soil microbial communities [19,20]. Planting indigenous sand-fixing plants, particularly drought-tolerant local legumes combined with straw checkerboard, represents an effective way to approach combat desertification and promote the formation of biological soil crusts [12,21,22,23]. A proven strategy in these areas is to use native, drought-tolerant pioneer plants such as *Caragana korshinskii*, a species that excels in the ecological restoration of sandy land.

Our most intriguing and novel finding is the specific and significantly higher relative abundance of the phylum Glomeromycota in soils under *C. korshinskii* stands (Cak and CakCos), as shown in Figure 3. Notably, while Ascomycota and Basidiomycota remained the dominant phyla across all treatments, which is a common pattern in soil fungal surveys [24,25], the relative abundance of the previously less abundant Glomeromycota emerged as the key differentiating feature. Furthermore, our multi-level taxonomic analysis (Figure 3 and Figure 4) provides a clear, hierarchical view of this enrichment. At the phylum level, Glomeromycota was significantly more abundant in *C. korshinskii* treatments; this pattern was consistently observed at lower taxonomic levels, including the order Glomerales, the family Glomeraceae, and the genera *Glomus* and *Septoglomus*. The phylogenetic tree (Figure 5) further confirmed that *Glomus* and its close relative *Rhizophagus* were the most prominently represented taxa within Glomeromycota. Importantly, the Glomeromycota phylum comprises a major group of fungi, primarily consisting of arbuscular mycorrhizal fungi (AMF). AMF play essential ecological roles in ecosystems, including nutrient uptake and transfer, modification of the physical soil environment, and regulation of plant interactions with other organisms [10]. AMF are typically natural symbionts of plant roots and significantly enhance nutrient availability and stress tolerance in their hosts [26]. Most studies on AMF have focused on the rhizosphere; conversely, their presence in non-rhizosphere soils has received limited research attention. This consistent pattern across all taxonomic levels supports the conclusion that stands containing *C. korshinskii* are associated with higher relative sequence abundance of Glomeromycota in the topsoil. However, because ITS amplicon sequencing cannot distinguish DNA originating from active living hyphae, dead residual DNA, or root-colonizing AMF, these results should be interpreted strictly as patterns of relative sequence abundance rather than as evidence of functional or biomass-based enrichment. Distinguishing among these sources should be a focus of future research.

To explore why *C. korshinskii* may be associated with Glomeromycota, we propose two non-mutually exclusive hypothetical mechanisms that could underpin this association; neither can be validated from our observational ITS dataset alone. First, according to the “plant-driven selection of microbes” hypothesis [27], plants can shape their rhizosphere microbiome through root exudates with distinct chemical profiles. *C. korshinskii* likely releases unique blends of flavonoids, strigolactones, or other signaling molecules that selectively chemo-attract and stimulate the germination and growth of *Glomus* and *Septoglomus* species [28]. Second, the altered soil environment created by *C. korshinskii* (higher SM, TC, TN, EC) may simply be more favorable for these AMF taxa. Our correlation analysis (Figure 6) strongly supports this point, showing significant positive correlations between Glomeromycota, *Glomus*, and *Septoglomus* with SM, TC, EC, and the C:N ratio. Glomeromycota, being obligate biotrophs, are highly dependent on their host plant for carbon [29]. Enhanced plant growth and carbon fixation by *C. korshinskii* likely increase carbon allocation to aboveground litter and roots, thereby supplying more carbon to AMF symbionts and fueling their growth and reproduction [30]. This enrichment is ecologically significant because Glomeromycota consist almost entirely of AMF. The establishment of AM symbiosis represents a key evolutionary adaptation that enabled plants to colonize terrestrial habitats. In desert ecosystem restoration, this symbiosis is not merely beneficial but essential for plant survival and growth under extreme stress [31,32]. However, these mechanisms remain hypotheses that require controlled experiments and direct measurements of AMF colonization, hyphal density, glomalin-related soil protein, and soil aggregation.

In the present study, *Glomus* and *Septoglomus* (Figure 4D,E and Figure 5) were identified as the predominant Glomeromycota taxa with higher relative abundance under *C. korshinskii*. AMF play vital roles in ecosystem functioning, including nutrient acquisition and transfer, soil structure improvement, and mediation of plant interactions with other organisms [10]. Typically forming natural symbioses with plant roots, AMF significantly enhance host nutrient availability and stress tolerance [26]. The enrichment of Glomeromycota is not a taxonomic curiosity but a functional milestone with direct consequences for the success of sand-fixation efforts. AMF, primarily from genera like *Glomus* and *Rhizophagus*, play multifaceted roles in ecosystem functioning, with enhanced nutrient acquisition being the most recognized benefit [33,34]. The growing evidence indicates that AMF can enhance the uptake of other nutrients like zinc, copper, and importantly, nitrogen in various forms [26], creating a positive feedback loop: *C. korshinskii* fixes N and enriches the soil, which supports AMF, which in turn improves the plant’s acquisition of N, leading to even better plant growth and further soil enrichment. Another key role of AMF lies in improved water relations: their hyphae can facilitate water uptake from soil micropores that are inaccessible to roots, especially as the soil dries [26]. The improved water status of AMF-colonized plants enhances their drought tolerance, a paramount trait in desert environments. The significant correlation found between Glomeromycota and SM may be bidirectional: the higher SM under *C. korshinskii* favors AMF, and the AMF, in turn, help the plant maintain water uptake, stabilizing the soil moisture regime [35].

Conversely, its presence in non-rhizosphere soils has received limited research attention, and our study breaks through this understanding. By moving beyond the macroscopic impacts of vegetation restoration, this research delves into the often-overlooked dynamics of soil microbial communities. We provide compelling evidence that sand-fixation using *C. korshinskii* specifically enriches Glomeromycota in the topsoil. This finding advances our understanding of the underlying mechanisms through which leguminous *Caragana* species contribute to reversing desertification.

### 3.3. The Functional Implications of AMF Enrichment for Ecosystem Restoration

The extraradical hyphae of AMF produce a glycoprotein called glomalin, which acts as a potent soil glue. Glomalin binds soil particles together, forming stable aggregates [10]. This aggregation is the very essence of sand fixation. It reduces wind and water erosion, increases soil porosity, and improves water-holding capacity. Therefore, if these sequence-based patterns are validated by direct measurements of fungal biomass, glomalin concentration and soil aggregation, the higher relative sequence abundance of Glomeromycota under *C. korshinskii* could contribute to the formation of a more stable soil structure, mitigating dune migration and sandstorm occurrence, which are the primary goals of the sand-fixation project [22,36]. However, sand fixation, glomalin, root colonization, and soil stability were not directly measured in this study.

AMF colonization can elicit systemic resistance in plants against soil-borne pathogens. By occupying root niches and consuming carbon sources, they can also effectively competitively exclude potential pathogenic fungi [10]. Our FUNGuild-based bioinformatic predictions (Figure 7) provide indirect and purely predictive support for this defensive role of AMF. Specifically, in *C. korshinskii*-associated treatments (Cak and CakCos), the predicted abundance of the Arbuscular Mycorrhizal (AM) guild was relatively higher and that of specific saprotrophic and potentially pathogenic guilds (e.g., Plant Pathogen/Undefined Saprotroph, PUS) was relatively lower compared to the Cof treatment. This shift in the fungal community’s functional balance toward more mutualistic taxa and away from potentially harmful groups fosters a healthier soil environment conducive to plant establishment [37,38]. In summary, the higher relative abundance of Glomeromycota may be a key mechanism through which *C. korshinskii* enhances its own fitness and accelerates ecosystem recovery. It is a biological tool that the plant employs to overcome multiple abiotic and biotic stresses, thereby consolidating the physical sand fixation achieved by the straw checkerboards.

Our study employed a multi-faceted approach to establish links between plant presence, soil properties, microbial community structure, and ecosystem function. The RDA (Figure 8) was particularly powerful in this regard, revealing that soil factors such as SOC, C:P ratio, TK, and SM were the primary drivers shaping the functional profile of the soil fungal community. Notably, despite distinct shifts in community composition and functional traits, the α-diversity of the overall fungal community remained unchanged (Supplementary Figure S1B). This finding indicates that *C. korshinskii* does not inherently enhance the total richness of fungal species but rather acts as a strong environmental filter, selectively promoting beneficial taxa such as Glomeromycota while suppressing others. This process is well-documented as a core driver of microbial community assembly in terrestrial ecosystems [39].

Consistent with the stable overall α-diversity observed in Section 2.2, this selective filtering represents a classic instance of deterministic selection shaping microbial communities, rather than non-specific enrichment of the entire microbial assemblage, which reinforces the fundamental concept of plant-specific microbiome assembly [40]. In the context of desert ecosystems, such plant-mediated filtering is particularly ecologically meaningful. Arid and semi-arid environments are characterized by harsh abiotic stresses, and microbial communities in these habitats are often structured by strong environmental filtering to favor stress-tolerant and mutualistic taxa [41]. Our results suggest that *C. korshinskii* amplifies this filtering effect by modulating soil properties (e.g., increasing SOC and SM), thereby creating a habitat niche that selectively favors Glomeromycota, which are taxa critical for sand fixation and plant stress resistance. This adaptive strategy not only enhances *C. korshinskii*’s own fitness in desert environments but also contributes to the stabilization of the surrounding soil ecosystem, highlighting the pivotal role of plant-microbe interactions in dryland restoration [42].

### 3.4. Limitations and Future Directions

While our study provides strong correlative evidence, it has certain limitations that point to fruitful avenues for future research. Regarding mechanistic insights, the field study identifies patterns and associations but cannot definitively prove causation, necessitating controlled greenhouse experiments (e.g., growing *C. korshinskii* in sterile soil inoculated with specific *Glomus* or *Septoglomus* isolates) to establish cause-and-effect relationships and elucidate the molecular dialogue (e.g., root exudate profiling) underlying this specific partnership. In terms of temporal dynamics, the 2020 snapshot study (on plantings from 2002) calls for chronosequence studies or long-term monitoring to reveal the assembly and evolution of the Glomeromycota community during ecosystem development, including when the enrichment manifests and its stability. For functional measurement, direct assessments of AMF functionality (e.g., hyphal density, glomalin-related soil protein (GRSP) concentration, and stable isotope tracing of nutrient flow via AMF hyphae) are needed to complement FUNGuild-based functional predictions. Additionally, the study’s focus on a specific region of the Mu Us Sandy Land means further testing is required to verify the generality of *C. korshinskii*’s effect on Glomeromycota across other desert ecosystems with different soil types, climates, and microbial species pools.

## 4. Materials and Methods

### 4.1. Study Area and Sampling Design

The study area was situated in the Baijitan National Nature Reserve in Lingwu, Ningxia. It is part of the core region for straw checkerboard combined shrub sand fixation on the southwestern edge of China’s Mu Us Sandy Land (37°58′28″ N, 106°24′06″ E). The study area exhibits a typical temperate continental climate characterized by drought and limited rainfall, high evaporation intensity, and uneven annual precipitation of approximately 255.2 mm. The majority of the precipitation is concentrated between July and September; moreover, the region experiences an annual average of 3080.2 h of sunshine, with a cumulative temperature range of daily averages ≥ 10 °C spanning from 2560.1 to 3334.8 °C. Additionally, there is a frost-free period lasting for 157d.

The area primarily consists of aeolian sandy soil with a sparse vegetation composition. Before 2001, the study area was severely affected by desertification. However, a straw checkerboard sand-fixing pattern (partially buried in sand) was implemented to address this issue. Then, indigenous shrubs dominated by *Caragana korshinskii*, *Corethrodendron fruticosum* var. *mongolicum*, *Calligonum mongolicum*, *C. scoparium*, and *Artemisia ordosica* were planted into the straw checkerboard in 2002. The sand-fixing patterns consisted of pure stand forests and two combinations of pairwise mixed forests [12]. It was observed that these sand-fixing patterns effectively mitigated dune migration and minimized the occurrence of sandstorms [22,36], especially the sand-fixation pattern involving *C. korshinskii*, which showed remarkable effect [4].

In the experimental area, we focused specifically on sand-fixation events involving *C. korshinskii*. Therefore, three sand-fixation patterns with a large planting area were selected: Cak (*C. korshinskii* pure shrub forest), Cof (*Corethrodendron fruticosum* var. *mongolicum* pure shrub forest), and CakCos (*C. korshinskii* and *C. scoparium* mixed shrub forest) as the subjects of our research. Among these three sand-fixation patterns, the one involving *C. korshinskii* included pure forest (Cak) and mixed pattern (CakCos), which stands in stark contrast to the other without *C. korshinskii* (Cof). Each sand-fixation pattern was represented by three independent observation areas (approximately 2 hm^2^), with a minimum distance of >2 km between areas. Within each observation area, five quadrats (10 × 10 m) were randomly placed along an S-shaped transect, and composite topsoil samples were collected from each quadrat. The observation area was treated as the experimental unit (*n* = 3 per pattern), and quadrats were considered subsamples.

### 4.2. Soil Sampling

In July 2020, we collected bulk topsoil (0–10 cm) after removing the surface organic layer and litter layer. The term “topsoil” in this study refers to bulk soil, not rhizosphere soil. Of the 45 collected samples, 43 were successfully sequenced; two samples from the Cak treatment were excluded due to insufficient DNA quality or sequencing failure. The final sample size was therefore 43 (Cak, *n* = 13; Cof, *n* = 15; CakCos, *n* = 15). The above collected samples were divided into two portions, and the SM content was determined immediately. One portion was placed in a sterile bag for analysis of soil physicochemical properties, while the other portion (approximately 20 g) was sieved through a 1 mm sieve, packed into a 10 mL sterile centrifuge tube, quickly transferred to a dry ice bucket, and subsequently transported back to the laboratory where it was stored at −80 °C for DNA extraction [43].

### 4.3. Soil Physicochemical Properties Analysis

The determination of SM involved weighing fresh and dried soil samples (dried at 105 °C until reaching a constant weight) during the sampling process. Subsequently, the air-dried soil samples were sieved through 10 and 100 mesh screens, while pH and EC were measured using a pH meter (PHS-3D, Shanghai, China) and a conductivity meter (DDS-307A, Shanghai, China), respectively. Soil TN, AN, TC, soil organic carbon (SOC), TK, available potassium (AK), total phosphorus (TP), and available phosphorus (AP) were determined following the methods described by Bao [44]. Specifically, AN was determined by the alkaline hydrolysis diffusion method, which measures readily hydrolyzable organic nitrogen and exchangeable ammonium; TN was determined by the Kjeldahl method; TC and SOC were measured by dichromate oxidation and elemental analysis, respectively; TP and AP were determined by molybdenum–antimony colorimetry; TK and AK were measured by flame photometry. All measurements were performed in triplicate.

### 4.4. Soil DNA Extraction, Amplification and Sequencing

Following the manufacturer’s instructions, fungal genomic DNA was extracted from approximately 0.5 g of sieved (1 mm) bulk topsoil using the FastDNA SPIN Kit (MP Biomedicals, Santa Ana, CA, USA). DNA was eluted in 100 μL of DES, and extracts were stored at −80 °C until PCR. The integrity and purity of the extracted DNA were assessed using agarose gel electrophoresis and a NanoDrop 2000 (Thermo Fisher Scientific, Waltham, MA, USA). Given that NanoDrop measurements may be affected by co-extracted humic substances and RNA, NanoDrop was employed solely as a rapid purity screening tool, whereas successful PCR amplification and sequencing served as the definitive criteria for quality assessment. Future studies should consider incorporating fluorescence-based quantification methods (e.g., Qubit or PicoGreen) to achieve more accurate DNA quantification. Subsequently, PCR amplification was conducted with the primer pair ITS1F (5′-CTTGGTCATTTAGAGGAAGTAA-3′) and ITS2 (5′-GCTGCGTTCTTCATCGATGC-3) [45]. The PCR reaction mixture (25 μL) contained 1× PCR buffer, 0.2 mM dNTPs, 0.4 μM each primer, 0.5 U Taq DNA polymerase, and approximately 10 ng template DNA. Thermal cycling conditions were: initial denaturation at 95 °C for 3 min; 30 cycles of 95 °C for 30 s, 55 °C for 30 s, and 72 °C for 30 s; and a final extension at 72 °C for 5 min. Paired-end sequencing (2 × 250 bp) was performed on the Illumina platform at Genesky Biotechnologies Inc. (Shanghai, China).

### 4.5. Sequence Processing

After performing quality control and filtering on the original data, we obtained high-quality sequencing data. QIIME2 (version 2023.5) with the DADA2 plugin was used for quality filtering, denoising, chimera removal, and merging of paired-end reads to generate amplicon sequence variants (ASVs). The resulting ASVs were subsequently clustered at 97% sequence similarity to generate operational taxonomic units (OTUs) for downstream taxonomic assignment, phylogenetic tree construction, and community statistical analyses. We clustered ASVs into 97% OTUs for downstream analyses to reduce noise from intragenomic ITS polymorphisms within Glomeromycota, which frequently produce minor sequence variants from single species. Taxonomy was assigned using the UNITE database (version 9.0) for fungal ITS sequences [46]. Fungal functional guild assignments were performed using the FUNGuild database (database version 1.1), which provides predictive annotations for fungal ecological groups [47,48]. Gaining insights into the abundance of these functional groups is crucial for comprehending soil functional processes. In our study, we investigated the fungi associated with sand-fixation patterns involving *C. korshinskii* and predicted the potential role of *C. korshinskii* in desert management by analyzing outputs from the FUNGuild database.

### 4.6. Statistical Analysis

The box diagram changes in physical and chemical properties and the differences in microbial groups at different classification levels of topsoil among each sand-fixation pattern were drawn based on column analyses with Kruskal–Wallis tests followed by Dunn’s post hoc test for multiple comparisons (nonparametric tests) using GraphPad Prism 9.0 (GraphPad Software, San Diego, CA, USA). For pairwise comparisons between Cak and Cof, and between CakCos and Cof, Mann–Whitney U tests were used. Bar charts for the composition of species, Venn diagrams, α-diversity, and correlation heatmaps were created using various packages (ggplot2, ggvenn, corrplot) in R (version 4.1.0). Redundancy analysis (RDA) was conducted to elucidate the relationship between soil factors and fungal functional groups using Canoco 5. Fungal functional-group abundance data were Hellinger-transformed prior to RDA to mitigate biases associated with compositional sequencing data. The difference in fungal groups between the sand-fixing treatments was assessed by employing linear discriminant analysis (LDA, log10) to identify significant differences in species effect values (i.e., |LDA| > 2 and *p* < 0.05). For visualization, the statistically filtered cladogram (|LDA| > 2 and *p* < 0.05) is shown in Figure 3; the exploratory cladogram without *p*-value filtering is provided in Supplementary Figure S3. The screening results were further analyzed using the LEfSe module at http://galaxy.biobakery.org/ to draw a cladogram (Galaxy version 1.0, accessed on 15 November 2024) [49]. The DNA sequences of OTUs at the species level were selected from Glomeromycota and aligned using MAFFT-7.037 [50], and subsequently utilized to construct a phylogenetic tree by MEGA X with 1000 bootstrap replicates [51]. The FUNGuild database annotates a more comprehensive fungal functional group, and its predictions link fungal taxa to ecological guilds [52]. Adobe Illustrator CS6 was used to complete the processing of each graphic panel, and statistical differences were expressed as *p* < 0.05, *p* < 0.01, and *p* < 0.001, respectively. *p*-values were corrected for multiple comparisons using the Benjamini–Hochberg false discovery rate (FDR) method where applicable.

## 5. Conclusions

Our observational comparison shows that *C. korshinskii* sand-fixation patterns are associated with higher topsoil Glomeromycota relative sequence abundance and distinct predicted fungal functional guilds. Cak and CakCos increased SM, EC, TN, and TC, and had higher Glomeromycota relative abundance, with *Glomus*, *Septoglomus*, and *Rhizophagus* as core taxa. FUNGuild-predicted outputs suggested a shift toward the AM guild and away from saprotrophic/potentially pathogenic guilds, with SOC, C:P, TK, and SM as associated environmental factors. These results are correlative and do not establish evidence for selective recruitment, in situ root colonization, or sand-fixation feedback. Neither enrichment patterns nor functional performance were directly measured in this study. This work generates hypotheses regarding the interactions between *C. korshinskii* and Glomeromycota in dryland restoration, provides supporting evidence for considering this shrub species in desertification-control practices, and offers perspectives for future research focusing on arbuscular mycorrhizal mycelial networks.

## Data Availability

The raw amplicon sequencing data generated in this study are available in the Genome Sequence Archive (GSA) of CNCB under BioProject accession number PRJCA068467.

## Supplementary Materials

The following supporting information can be downloaded at: https://www.mdpi.com/article/10.3390/plants15213338/s1. Table S1. Sequencing read statistics and quality control for the 43 topsoil samples across the three sand-fixation patterns; Figure S1. (A) Fungal community composition at the genus level. (B) Alpha diversity indices (ACE, Chao1, Shannon, and Simpson) of the fungal community. “ns” indicates no significant difference (Kruskal-Wallis test, *p* > 0.05); Figure S2. Linear discriminant analysis (LDA) scores of differentially abundant fungal taxa. The length of each bar represents the effect size; Figure S3. LEfSe cladogram showing the phylogenetic distribution of differentially abundant taxa (LDA > 2, without *p*-value filtering); Figure S4. Comparisons of fungal relative sequence abundance across all hierarchical taxonomic levels among treatments (a: phylum, b: order, c: family, d: genus, e: species). Significance: ns, *p* < 0.05, * *p* < 0.01, ** *p* < 0.001.
